# 18例十二指肠型滤泡性淋巴瘤的临床病理学研究

**DOI:** 10.3760/cma.j.cn121090-20230915-00125

**Published:** 2024-01

**Authors:** 艳茹 杜, 佳 李, 少祥 李, 春艳 关, 洪利 李, 子芬 高, 雪 李, 格红 董

**Affiliations:** 首都医科大学附属北京天坛医院病理科，北京 100070 Department of Pathology, Beijing Tiantan Hospital, Capital Medical University, Beijing 100070, China

## Abstract

为研究十二指肠型滤泡性淋巴瘤（duodenal-type follicular lymphoma，D-FL）的临床及病理学特点，总结2020年1月至2023年7月期间首都医科大学附属北京天坛医院病理科会诊及本院确诊的18例D-FL患者的临床症状、内镜特点、病理形态学特点、免疫表型、分子病理特征以及治疗随访经过。18例D-FL患者，男10例，女8例，中位年龄为49（32～69）岁。患者多为胃肠镜检查发现或出现常见胃肠道症状，如胃痛、反酸、呕吐及腹泻。内镜大多表现为多发灰白色小息肉样隆起。病理形态学上为黏膜层及黏膜下层可见淋巴滤泡样结构。免疫表型为肿瘤细胞广泛强表达CD20及BCL2，增殖活性低。免疫球蛋白克隆分析显示1例IgK单克隆重排（1/1），FISH检测1例BCL2基因重排（1/3）。所有患者均未进行靶向及化疗，采取等待及观望策略。中位随访12（2～34）个月。研究显示D-FL属于惰性淋巴瘤，好发于十二指肠，预后良好。

## Abstract (en)

To investigate the clinical and pathological characteristics of duodenal-type follicular lymphoma (D-FL), and to deepen the understanding of Duodenal-type follicular lymphoma. The clinical symptoms, endoscopic features, pathologic features, immunophenotype, molecular pathological features and treatment follow-up of 18 D-FL patients diagnosed in Department of Pathology, Beijing Tiantan Hospital affiliated to Capital Medical University between January 2020 and July 2023 were summarized. A total of 18 patients with D-FL were included, including 10 males and 8 females. The median age was 49 (32–69) years respectively. Most of the patients were found during gastroenteroscopy or presented with the common gastrointestinal symptoms of stomach pain, acid reflux, vomiting and diarrhea. Most endoscopic findings were multiple small gray and white polyposis. In the pathological morphology, the mucous layer and submucous layer showed lymphoid follicular structures with full and strained follicles. The immunophenotype showed that the tumor cells strongly expressed CD20 and BCL2 and had low proliferation activity. Immunoglobulin clonal analysis of 1 case showed IgK monoclonal rearrangement (1/1). FISH showed 1 case of BCL2 gene rearrangement (1/3). All patients did not receive targeted chemotherapy and adopted a wait-and-see strategy. Median follow-up was 12 (2–34) months. This study shows that D-FL is an indolent lymphoma, which tends to occur in the duodenum and has a good prognosis.

十二指肠型滤泡性淋巴瘤（duodenal-type follicular lymphoma，D-FL）是2016版淋巴造血组织肿瘤WHO分类中新纳入的一种滤泡性淋巴瘤（FL）的特殊亚型，通常情况下主要发生于十二指肠降段，但也可能累及远端小肠，而不累及淋巴结，具有其独特的临床、病理和生物学特征[1]。2019 版WHO消化系统肿瘤最新分类中继续描述了这个亚型。Takata等[2]发现D-FL的病理形态学有独特的特点，与普通的淋巴结滤泡性淋巴瘤不同，D-FL的肿瘤性滤泡内缺少滤泡树突细胞网络，而呈现滤泡边缘浓集的现象。D-FL预后较好，但综合国内外文献分析，该病有转化为系统性淋巴瘤的病例报道。针对不同研究对该病的认识仍然存在争议。国内外有关D-FL大宗病例报道较少。本文总结18例D-FL患者的临床表现、内镜特点、病理形态学、免疫表型、克隆分析及BCL2基因断裂重排情况，并进行临床随访。

## 病例与方法

1. 患者入组情况：本研究纳入首都医科大学附属北京天坛医院病理科2020年1月至2023年7月本院及会诊确诊的D-FL患者18例，电话随访患者，末次随访时间是2023年9月18日。D-FL入组标准：①内镜特点、病理形态学及免疫表型符合D-FL；②询问病史并进行PET-CT检查除外了全身病变累及十二指肠或回肠。

2. 免疫组化：内镜活检标本经10％中性福尔马林固定，常规脱水，石蜡包埋切片，苏木素-伊红染色法（HE染色）。免疫组织化学染色采用EnVision两步法，所用一抗试剂CD20、CD3、CD5、CD10、CD21、CD23、BCL2、BCL6、CyclinD1购自北京中杉金桥生物技术开发有限公司，Ki-67及PAX-5购自瑞士罗氏公司。使用罗氏和徕卡全自动免疫组织化学染色仪进行染色。

3. 克隆分析及FISH：本组中1例患者进行了免疫球蛋白克隆重链及轻链重排分析，检测仪器使用ABI 3500基因测序仪，检测方法包括DNA提取，PCR和毛细管电泳。DNA质量评估合格，片段长度为400 bp，DNA浓度为197.5 ng/µl；1例患者采用BCL2基因断裂探针进行杂交，探针购自美国雅培公司，实验操作按照试剂盒说明书进行。

## 结果

1. 临床指标：18例D-FL患者，男10例，女8例，中位年龄为49（32～69）岁。18例患者中有8例为胃肠镜检查时偶然发现，不伴有特殊症状；2例十二指肠降部D-FL患者表现为反酸、胃部烧灼感；2例十二指肠降部D-FL患者表现为胃肠不适；1例十二指肠降部D-FL患者表现为呕吐；1例回肠末端D-FL患者表现为稀便；1例回肠末端D-FL患者表现为腹痛；1例因既往有结肠腺瘤病史，复查肠镜检查时偶然发现。

2. 内镜表现：内镜下表现为多发小息肉或小斑块样隆起，直径1～5 mm，灰白色，未见出血及坏死。患者病变部位主要位于十二指肠降部，位于回肠者，病变位于回肠末端（图1）。

**图1:**
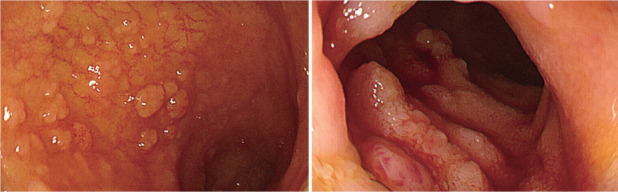
十二指肠型滤泡性淋巴瘤患者内镜下回肠末端病变 注 内镜下回肠末端黏膜可见多个大小不一灰白色息肉样隆起

3. 形态学表现及免疫组化结果：黏膜层及黏膜下层可见淋巴滤泡增生，淋巴滤泡由生发中心细胞组成，增生细胞形态单一，缺少“星空”现象，套区不明显。肿瘤细胞体积小至中等，胞质少，核不规则，可见核分裂，核仁不明显。偶见中心母细胞，<10/HPF（图2A）；18例D-FL肿瘤细胞均表达CD20、PAX-5、CD10、BCL6及BCL2。CD20（图2B）及PAX-5表达证实肿瘤细胞为B细胞，CD10和BCL6阳性支持肿瘤细胞来源生发中心细胞（图2C D）。BCL2阳性支持滤泡中心细胞为肿瘤细胞（图2E）。CD21（图2F）和CD23显示肿瘤细胞的滤泡树突状细胞（FDC）网在生发中心缺失呈斑驳状，而边缘浓集。肿瘤细胞不表达CD3（图2G）、CD5、CyclinD1，可以除外T细胞淋巴瘤、慢性淋巴细胞白血病及套细胞淋巴瘤。肿瘤细胞增殖活性低，Ki-67增殖指数<10％（图2H），提示肿瘤细胞生长缓慢，生物学形态学为惰性。

**图2:**
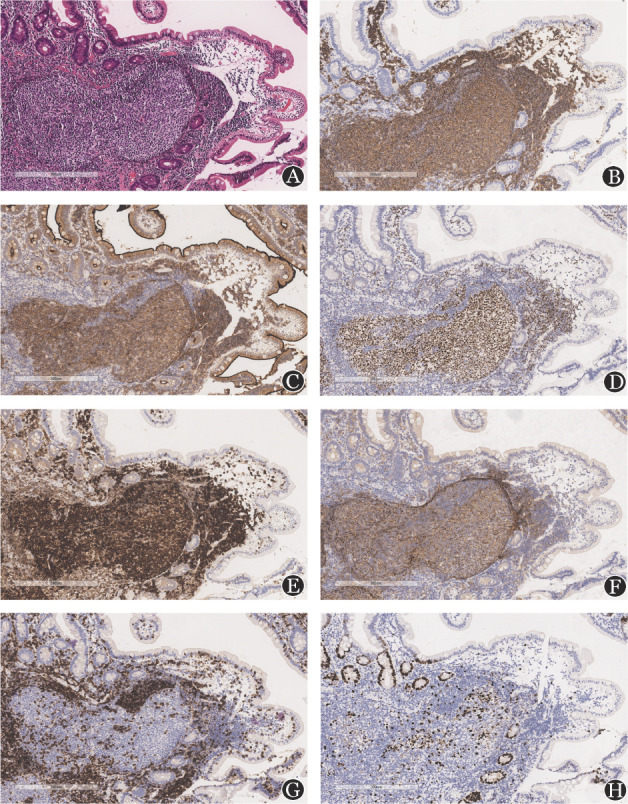
十二指肠型滤泡性淋巴瘤患者的苏木素-伊红染色及免疫组化（EnVision法）表达（×100） **A** 十二指肠黏膜内可见淋巴组织增生，呈滤泡结构；**B** CD20染色：肿瘤细胞广泛强表达CD20；**C** CD10染色：肿瘤细胞表达CD10；**D** BCL6染色：肿瘤细胞表达BCL6；**E** BCL2染色：肿瘤细胞表达BCL2；**F** CD21染色：显示肿瘤细胞位于滤泡树突状细胞（FDC）网内，FDC网在生发中心处呈斑驳改变，边缘浓集；**G** CD3染色：肿瘤细胞不表达CD3；**H** Ki-67染色：肿瘤细胞增殖指数为10％

4. FISH与免疫球蛋白重链和轻链克隆分析结果：3例患者进行了BCL2 FISH检测，其中1例发现BCL2基因断裂重排（图3A）；1例患者进行了免疫球蛋白重链和轻链克隆分析，该患者存在免疫球蛋白轻链重排（图3B），支持为肿瘤性病变。

**图3:**
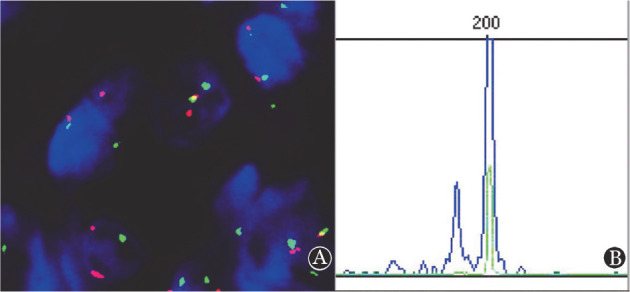
十二指肠型滤泡性淋巴瘤患者的FISH与免疫球蛋白重链和轻链克隆分析结果 **A** FISH检测显示BCL2基因断裂重排；**B** IG基因重排克隆性分析显示IGK TubeB为克隆性重排（200 bp）

5. 治疗、预后与随访：本组病例均未进行R-CHOP（利妥昔单抗、环磷酰胺、多柔比星、长春新碱及泼尼松）方案化疗或利妥昔单抗单药靶向治疗，中位随访时间为12（2～34）个月，目前随访时间最长的患者达34个月，病情稳定，未发现进展。3例患者失访。

## 讨论

本文总结了18例D-FL的临床症状、内镜特点、病理形态学特点、免疫表型、分子病理特征以及治疗随访经过。D-FL生物学行为介于FL和黏膜相关淋巴组织结外边缘区B细胞淋巴瘤（mucosa associated lymphoid tissue，MALT）之间[3]–[5]。患者发病年龄为中年，无性别分布差异[6]。肿瘤组织由形态单一的中心细胞组成，中心母细胞罕见，与淋巴结FL 1～2级的病理学表现相似。其免疫表型也与淋巴结FL类似，肿瘤细胞表达CD20、CD10、BCL6及BCL2，通常情况下细胞增殖活性低。FDC通常局限在肿瘤性滤泡内部的外围处。有研究发现与其他FL相比，D-FL缺乏FDC；此外，与其他FL相比其有免疫球蛋白重链基因差异，其表现与MALT淋巴瘤相似[2]。一些基础研究发现FL细胞在肿瘤内与FDC相互作用，并且FDC的存在为淋巴瘤细胞提供了生长优势[7]–[8]。因此FDC的缺失可能导致十二指肠FL的组织学分级较低，肿瘤进展较慢[2]。约83％的D-FL病例显示肿瘤细胞携带t（14;18）（q32;q21）（IGH::BCL2），该特点与淋巴结FL一致[6]。然而与淋巴结FL不同的是，D-FL肿瘤细胞染色体1p缺失相对常见，该区域携带TNFRSF14基因[6]。D-FL肿瘤细胞伴有IGH基因的体细胞超突变，因而推测该肿瘤细胞可能起源于记忆性B细胞[6]。Tari等[9]进行的随机对照研究共纳入29例D-FL，其中14例采用利妥昔单抗治疗及化疗，而另一组15例患者采用观望及等待的方式，两组的总生存率均为100％，无进展生存率差异无统计学意义。国外的D-FL回顾性研究显示D-FL预后较好，多采用观察和等待策略[6],[10]–[12]。国内学者的回顾性研究显示DF-L患者采取观察的方案未进一步靶向治疗及化疗者，病情稳定，未发现进展[13]–[17]。然而国内张芬等[18]的研究显示4例D-FL，3例采用利妥昔单抗治疗后，病变完全消退，随访27～42个月，未见复发，另外1例在确诊后采取等待观望方案，15个月后进展为系统性淋巴瘤，其Ki-67指数为15％。最新的研究[19]发现在随访超过10年的5例D-FL患者中，2例患者出现骨髓累及，经过利妥昔单抗及放射治疗后再次获得完全缓解。其余3例中，1例化疗后复发，再次化疗后获得完全缓解；1例转化为弥漫性大B细胞淋巴瘤，全身播散；1例虽经化疗最终死于该病，死亡时间距发病21年。但是该研究未提及这几例复发进展的病例在形态学特点及免疫表型与未出现复发进展的病例有何不同。有研究[12]进行多因素分析显示，女性、未出现腹部症状及未累及十二指肠降段与较好的预后有关。由于大多数关于D-FL的研究都是回顾性的，因此无法推断出最佳治疗的确切建议[6],[9],[11],[20]。通常，D-FL使用四种治疗策略，包括等待观望策略、放射治疗、免疫疗法和免疫化学疗法，并且每种策略的有效性都有报道[6],[11]–[12],[20]。诊断D-FL，需要鉴别淋巴结低级别FL累及十二指肠、MALT淋巴瘤和套细胞淋巴瘤：①淋巴结低级别FL累及十二指肠：常有淋巴结FL的病史，全身淋巴结肿大，累及十二指肠管壁全层，而不是仅局限于黏膜层和黏膜下层。②MALT淋巴瘤：肿瘤早期常围绕淋巴滤泡，浸润于边缘区，边缘区范围逐渐扩大，最后相互融合成片，并取代部分或全部滤泡（滤泡植入）。CD20和CK标记可清楚显示淋巴上皮病变。③套细胞淋巴瘤：肿瘤可随病变发展呈套区增宽型、结节型和弥漫型3种结构。肿瘤细胞形态较为一致，核小至中等大，核染色比小淋巴细胞浅，核型多不规则。可见血管壁玻璃样变性及散在分布单个的上皮样组织细胞，呈“满天星”样。肿瘤细胞特征性表达CD5、CyclinD1和SOX-11，常携带CCND1基因易位，而不携带BCL2基因易位。因此需综合临床病史、内镜改变、组织病理学、免疫表型及分子遗传学特征来鉴别D-FL与其他淋巴瘤，明确诊断。

总之，D-FL属于惰性淋巴瘤，好发于十二指肠，预后相对较好，主要的处理方式仍为随访观察。能够给临床以提示，避免过度治疗。本研究随访时间短，病变长期预后尚未可知。后续需要对患者继续随访，同时纳入更多的D-FL患者，详细观察其临床病理学及分子特征。
